# Innervation pattern of the unclosed detrusor muscle in classic bladder exstrophy: a study of patients with urothelial overexpression of nerve growth factor

**DOI:** 10.1007/s00383-024-05649-5

**Published:** 2024-03-05

**Authors:** Martin Promm, Wolfgang Otto, Stefanie Götz, Maximilian Burger, Karolina Müller, Peter Rubenwolf, Winfried L. Neuhuber, Wolfgang H. Rösch

**Affiliations:** 1 Department of Pediatric Urology, Clinic St. Hedwig, University Medical Center of Regensburg, Steinmetzstr. 1-3, 93049 Regensburg, Germany; 2 Department of Urology, University of Regensburg, Caritas St. Josef Medical Center, Landshuter Str. 65, 93053 Regensburg, Germany; 3 Center for Clinical Studies, University Medical Center Regensburg, Franz-Josef-Strauss-Allee 11, 93053 Regensburg, Germany; 4 Department of Urology, University Medical Center Frankfurt, Theodor-Stern-Kai 7, 60590 Frankfurt, Germany; 5 Institute of Anatomy and Cell Biology, Friedrich-Alexander University Erlangen-Nürnberg (FAU), Universitaetsstr. 19, 91054 Erlangen, Germany

**Keywords:** Bladder exstrophy-epispadias complex, Immunohistochemistry, Nerve growth factor, Urothelium, Delayed procedure

## Abstract

**Purpose:**

An overexpression of nerve growth factor (NGF) in the urothelium is discussed to lead to neuronal hyperinnervation of the bladder detrusor. The aim was to assess the sensory and sympathetic innervation of the detrusor in unclosed exstrophic bladders patients with known overexpression of NGF in the urothelium.

**Methods:**

Full-thickness bladder biopsies were prospectively obtained from 34 infants at delayed primary bladder closure between 01/2015 and 04/2020. The bladder biopsies were immunohistochemically stained with antibodies against S100, calcitonin gene-related peptide (anti-CGRP), Neurofilament 200 (anti-NF200), and tyrosine-hydroxylase (anti-TH). Specimens from 6 children with congenital vesicoureterorenal reflux (VUR) served as controls.

**Results:**

There was no statistically significant difference in nerve fiber density in any of the immunohistochemical assessments (anti-S100 [*p* = 0.210], anti-CGRP [*p* = 0.897], anti-NF200 [*p* = 0.897]), and anti-TH [*p* = 0.956]) between patients with BE and patients with VUR. However, we observed a trend toward lower nerve fiber densities in exstrophic detrusor.

**Conclusion:**

Overall our results showed an unharmed innervation pattern in this cohort but a lower density of nerve fibers in the detrusor compared to controls. Further studies in patients after successful primary closure are needed to clarify the potential impact of the urothelial overexpression of NGF modulating the innervation pattern in exstrophic bladders.

## Introduction

Several concepts for the primary closure of classic bladder exstrophy (BE) are available, differing in approaches, the approximation of the symphysis with and without osteotomy, and the timing of surgical intervention [1]. Irrespective of the mode of reconstruction, remarkable interindividual differences exist in the development of capacity, compliance, and detrusor function. The reasons for this variability in the outcome seem complex. Alteration of bladder wall components, including urothelium, but also alteration of the innervation pattern, has been discussed in this context [2–6].

In healthy people, the functional urinary bladder stores urine as a low-pressure reservoir and can be voluntarily emptied; for this, a healthy nerve supply is assumed. Innervation is made up of sensory and autonomic neurons. Both components are essential for its physiologic function. Sensory nerves are distributed uniformly in the detrusor. Mechanosensitive afferent fibers, usually myelinated Aδ fibers, transmit information about the normal sensation of bladder filling to the central nervous system. Most of the unmyelinated afferent C fibers are quiescent during normal filling and are usually responsible for nociception. So, afferents are important for the generation of storage and micturition reflexes and for noticing bladder damages. Motor innervation of the detrusor is regulated by sympathetic and parasympathetic neurons, their interaction coordinates the storage and void phases to allow continence and complete bladder emptying [8–10].

We have previously investigated histological alterations and distribution of the nerve growth factor receptor p 75 (NGFR p75) in the unclosed exstrophic bladder wall. In addition to significant histologic changes with regard to acute inflammation, squamous metaplasia, and cystitis glandularis, we found a statistically significant increase in the occurrence of NGFR p75 in the urothelium compared to the control group with patients with congenital vesicoureterorenal reflux [5]. In transgenic mice, chronic overexpression of urothelial NGF (nerve growth factor) has been shown to lead to neuronal proliferation of sensory and sympathetic nerve fibers of the detrusor that mediate changes in bladder function, including increased urinary bladder reflex activity and hyperalgesia in response to inflammation or tissue injury [11].

The results of our previous study stimulated the present evaluation to assess the innervation pattern of an unclosed exstrophic bladder wall in patients with NGF urothelial overexpression to extend previous studies on the topic of bladder differentiation changes in patients with classic exstrophy patients. [6, 7, 12, 13].

### Patients and methods

Full-wall transmural bladder biopsies from the posterior bladder wall of patients with classic BE were prospectively obtained at the time of delayed primary bladder closure, which includes repair of the epispadias and inguinal hernias in boys, as well as approximation of symphysis. This study only included patients with classic BE and excluded other entities within the bladder-exstrophy-epispadias complex (BEEC). Specimens from children with congenital vesicoureterorenal reflux (VUR) served as controls because primary VUR does not cause major bladder alterations, and this entity has been repeatedly used as a control group in studies of BE [5, 6, 14–18].

The collection of samples and the immunohistochemical evaluation were approved by the Ethics Committee of the University of Regensburg, and written informed consent was obtained from the parents.

### Immunohistochemistry

Biopsies were fixed in 4% formalin, embedded in paraffin, and sectioned at 4 μm. These sections were mounted onto poly-L-lysine-coated glass slides and incubated in a BenchMark IHC Full System immunostainer (Roche Diagnostics, Mannheim, Germany) for immunohistochemistry. According to the manufacturer’s instructions, we used the avidin–biotin peroxidase method with diaminobenzidine as chromogen (UltraView DAB Detection Kit, Firma Roche Diagnostics, Mannheim, Germany) and hematoxylin as counterstain. Bladder biopsies were immunohistochemically stained with antibodies against S100, Calcitonin gene related peptide (anti-CGRP), neurofilament 200 (anti-NF200) and anti-tyrosine-hydroxylase (anti-TH) to determine the following neuronal subtypes (Table 1).

- S100 to assess nerve fibers in general,
- NF200 to assess afferent myelinated Aδ sensory fibers; NF200 also stains intrinsic autonomic neurons in the efferent,
- CGRP to assess afferent peptidergic fibers, many of them putative nociceptors, and
- TH to assess efferent catecholaminergic and largely sympathetic nerve fibers.

**Table 1:** Antibodies used for dilution

Antibody	Dilution	Company
Anti-CGRP rabbit polyclonal	1:1500	Dianova, Germany
Anti-S100 rabbit polyclonal	1:1000	Agilent, USA
Anti-NF200 mouse monoclonal	1:200	Sigma, Germany
Anti-TH rabbit polyclonal	1:75	Abcam, UK

Table 1 shows the primary antibodies and their dilution. All specimens were semiquantitatively independently evaluated by a neuroanatomist (WLN) using a light microscope (Leica Aristoplan, Leica, Bensheim, Germany) and by a pediatric urologist (MP) using a Zeiss Lab A1 microscope (Zeiss, Jena, Germany). The density of the markers was classified as absent, sparse, moderately dense or dense. The observations in patients with BE were compared to those of the control group.

### Statistical analysis

Statistical analysis was performed using the SPSS software package SPSS (Version 26, SPSS Inc, Chicago, Illinois). Descriptive analyses were performed using frequency (*n*), percentage (%), mean (m) and standard deviation (± SD). The Mann–Whitney U test was used to compare S100, NF200, CGRP, and TH between the two groups of patients. The level of significance was established at *p* < 0.05 for all tests. Data analyses were conducted in an exploratory manner; therefore, no adjustments were made for multiple tests.

## Results

### Patient data

Between January 2015 and April 2020, 39 patients with BE were referred to our department for delayed primary closure. 5 parents had refused to give their consent on behalf of their child for participation in this study. Thus, biopsies were prospectively obtained from 34 infants (20 boys and 14 girls) with BE, who underwent primary bladder closure. The mean age at the time of surgery was 61 ± 23.9 days (min = 38, max = 169). Bladder biopsies from six children (two boys and four girls) with VUR serve as controls. The mean age at the time of surgery was 412 ± 292.4 days (min = 8, max = 822). All these patients had primary higher-grade reflux (III–IV). None of them suffered from a neurologic disorder or showed clinical signs of bladder dysfunction or abnormalities typical of bladder dysfunction on voiding cystourethrogram.

### Immunohistochemical analysis

**Fig. 1:**
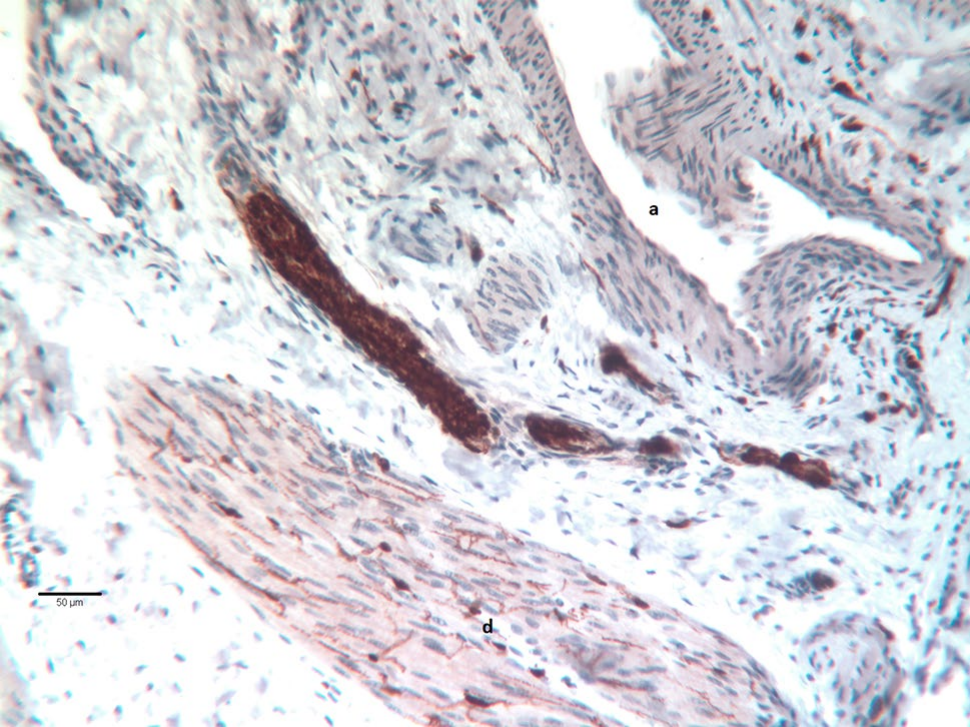
Bladder wall of a girl aged 116 days at primary closure. Immunohistochemistry for S100 shows a thick nerve fiber bundle and smaller bundles in the adventitia of a small artery (a). Note the single varicose axons between the smooth muscle fibers of a detrusor bundle (d)

**Fig. 2:**
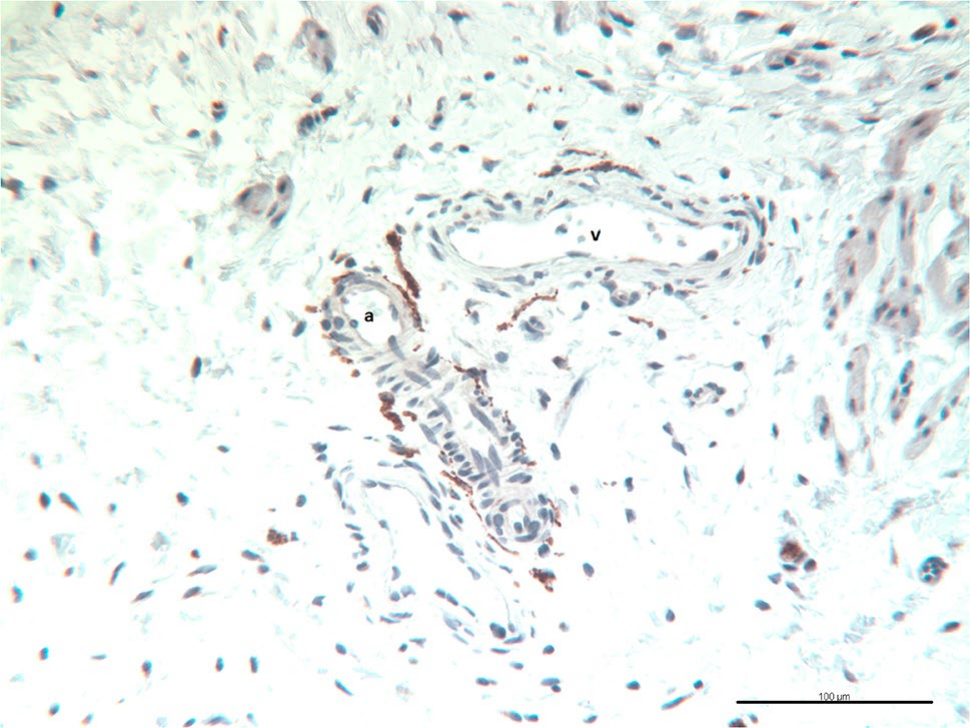
Bladder wall of a girl aged 62 days at primary closure. An arteriole (a) and a venule (v) in a connective tissue septum of the detrusor are in close contact to TH immunohistologically stained varicose axons. Note the higher fiber density around the arteriole

**Fig. 3:**
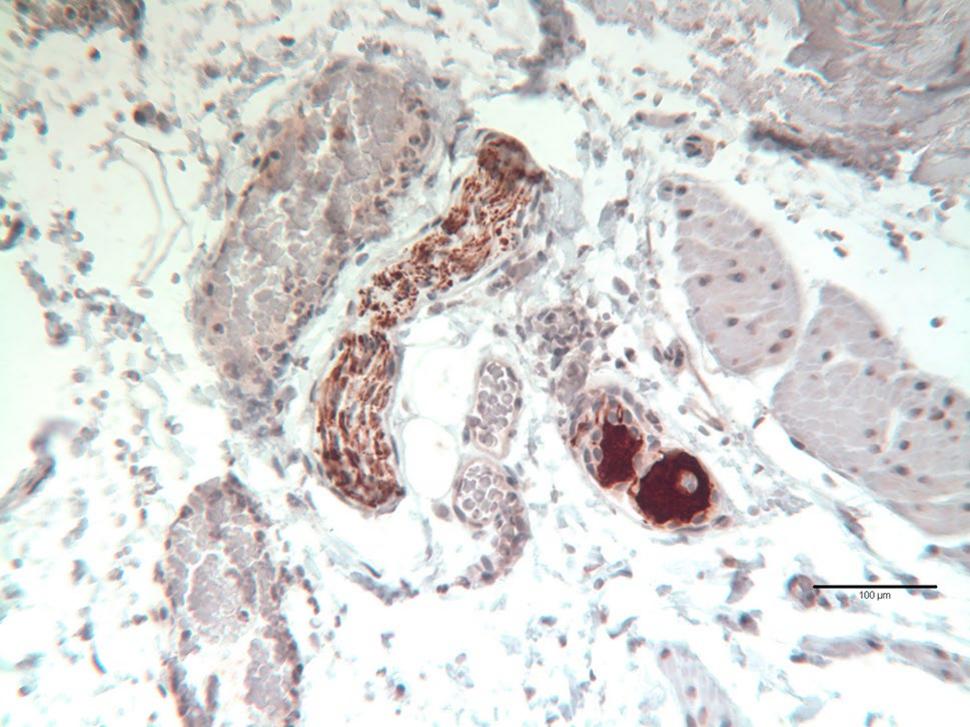
Bladder wall of a girl aged 64 days at primary closure showing a large bundle of NF200 immunoreactive axons. A small ganglion contains NF200 positive neuronal cell bodies

**Fig. 4:**
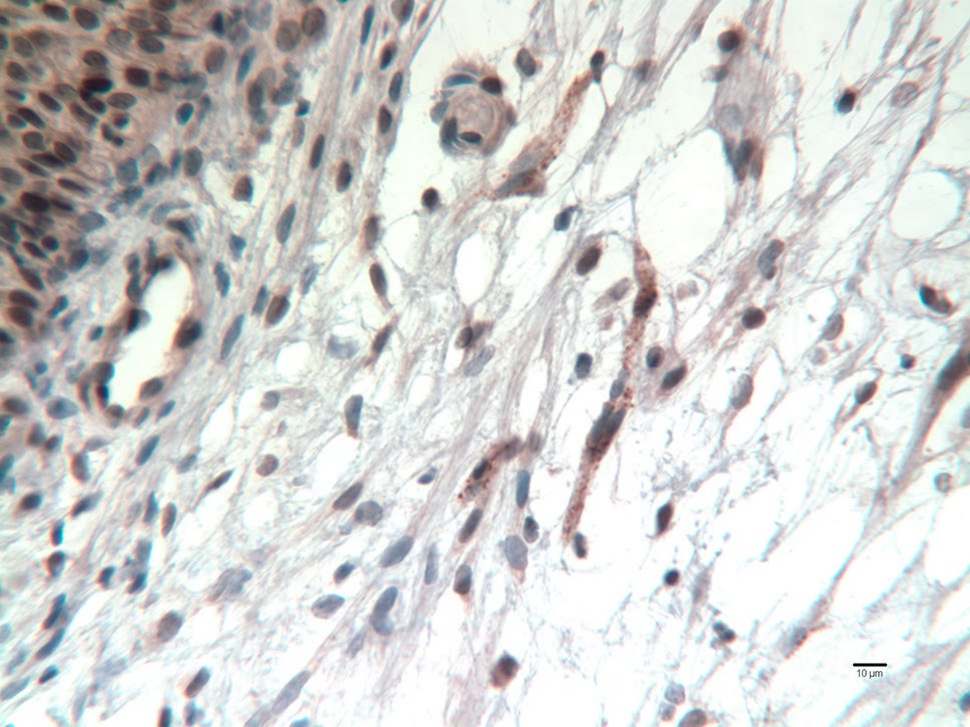
Bladder wall of a girl aged 62 days at primary closure, showing three small nerve fiber bundles staining for CGRP (fine brown granules) beneath the urothelium (upper left corner)

Immunostaining for S100, NF200, CGRP, and TH was observed in large and small nerves between the smooth muscle fiber bundles of the detrusor and in the lamina propria of the mucosa. No intraepithelial nerve fibers could be detected. As a general neuroglial marker, immunoreactivity for S100 was detected in nerve fiber bundles in all areas and was also regularly found between single smooth muscle fibers of the detrusor (Fig. 1). This finding was in strong contrast to TH, which was concentrated in perivascular fibers and varicosities (Fig. 2) and only occasionally observed within the detrusor muscle itself. NF200 was also found in nerves of various sizes without any predilection for a particular location and in intrinsic ganglion neurons (Fig. 3). CGRP immunostaining showed very fine fibers in the perivascular and lamina propria bundles (Fig. 4). In some places, intrinsic ganglion neurons were encircled by CGRP-positive varicosities (Fig. 4). Unfortunately, available antibodies to cholinergic markers (choline acetyltransferase and vesicular acetylcholine transporter) did not work well on our material. Because TH immunolabeling indicative of sympathetic axons was rare or absent from the detrusor muscle itself, S100-positive varicose fibers between its muscle fibers may represent cholinergic innervation (Fig. 1).

Although the comparison between BE patients (*n* = 34) and VUR patients (*n* = 6 showed a lower density of nerve fibers in the detrusor muscle, the density of S100, NF200, CGRP and TH did not differ significantly between the two groups (*p*-values > 0.050). These observations are summarized in Table 2.

**Table 2:** Differences in immunostaining

		Absent	Sparse	Moderately dense	Dense	*p*-value
S100	Patients with BE	0 (0)	15 (44.1)	10 (29.4)	9 (26.5)	0.210
	Patients with VUR	0 (0)	1 (16.7)	2 (33.3)	3 (50.0)	
NF200	Patients with BE	10 (29.4)	15 (44.1)	5 (14.7)	4 (11.8)	0.197
	Patients with VUR	1 (16.7)	1 (16.7)	3 (50.0)	1 (16.7)	
CGRP	Patients with BE	20 (58.8)	7 (20.6)	3 (8.8)	4 (11.8)	0.897
	Patients with VUR	3 (50.0)	2 (33.3)	1 (16.7)	0 (0)	
TH	Patients with BE	3 (8.8)	15 (44.1)	10 (29.4)	6 (17.6)	0.956
	Patients with VUR	0 (0)	2 (50.0)	3 (50.0)	0 (0)	

Observation of S100, NF200, CGRP, and TH in biopsies obtained from 34 patients with bladder exstrophy (BE) during primary closure and from 6 patients with congenital vesicoureterorenal reflux (VUR) during reflux surgery who served as controls. The table shows the frequency and, in brackets, the percentage

## Discussion

Successful initial bladder closure in patients with BE is one of the cornerstones to achieve continence and prevent damage to the upper urinary tract [19, 20]. In addition to histological changes in the bladder wall, innervation is thought to be important for further bladder development [21]. The overexpression of NGF in the urothelium is discussed to lead to neuronal hyperinnervation. Therefore, we assess sensory and sympathetic innervation using S100, NF200, CGRP and TH of the detrusor in patients with unclosed exstrophic bladders with known urothelial overexpression of NGF. The findings were compared between 34 BE patients who presented for delayed primary closure and 6 VUR patients who served as controls. Our investigation did not yield any significant differences in the parameters evaluated between these two groups.

In 1997, Rösch et al. analyzed biopsies obtained from 22 patients with the bladder-exstrophy-epispadias complex (BEEC) during open surgery and compared the innervation pattern with that of biopsies from 19 patients from postoperative urodynamic studies [7]. Biopsies from six healthy children of similar age served as control. Only 4 of the 22 patients had been referred to BE for primary delayed closure and 8 of the 22 patients after a failed BE reconstruction. The other 10 patients had isolated epispadias, cloacal exstrophy, or transition forms. Indirect immunohistochemistry was performed for CGRP, vasoactive intestinal polypeptide (VIP), neuropeptide Y (NPY), and substance P (SP). Samples were subjectively assessed with immunofluorescence by three independent investigators. All cloacal exstrophies and transition forms showed a pathologic innervation pattern with noticeable caliber differences in nerve fibers and bundles, also with increased innervation density. Patients with failed exstrophy reconstruction showed innervation deficiencies in the detrusor muscle but an increased innervation pattern intraepithelially and subepithelially. No morphologic abnormal innervation of these markers was found, neither in the four patients with isolated epispadias nor in the four patients with BE at delayed primary closure [7].

In 2007, the same group published their findings on characteristic alterations in the distribution pattern of muscarinic receptors on the exstrophic bladder walls [17]. 33 patients with BE were included in that study, of whom 10 had presented for primary closure and the other 23 patients with bladder dehiscence or persistent incontinence. Four children with VUR and four organ donors of the same age group served as the control group. The samples were subjectively assessed by three independent investigators. Among the different known M receptors (muscarinic receptors), especially the subtypes M2 and M3 receptors are present in the human bladder wall, and M3 receptors are predominantly responsible for the contraction of the detrusor muscle. The authors showed that most M2 receptors (80%) but not M3 receptors (20%) are present in the bladder wall. The distribution and density of M2-receptors in healthy bladders and exstrophic bladders for primary closure seemed identical. M2 receptors are typically arranged circularly around smooth muscle fibers. Patients with multiple bladder surgeries showed a reduced density of M2-receptors, as well as a heterogeneous distribution pattern. In contrast to M2-receptors, the density of M3-receptors was considerably higher and showed a different distribution pattern, although the difference was not significant. The M3 receptor in healthy bladders was characteristically located in the periphery of smooth muscle fibers in contrast to the intramuscular location in exstrophic bladders (operated and unoperated) [17].

In 1999, Mathews et al. examined the innervation pattern of the bladder in infants with BE [12]. In their study, biopsies of the anterior bladder wall of 10 infants with BE aged 1 to 90 days were taken at primary closure and compared to 10 bladder biopsies of newborns who had died of cardiac causes. All samples were evaluated for immunohistochemical staining of S100 only. The entire tissue section was assessed using a morphometric system. The results showed that myelinated and in particular small-caliber nerve fibers were significantly reduced in biopsies of patients with BE. In contrast to our findings, Mathews et al. postulated a statistically significant reduction in myelinated nerve fibers in newborns with BE, possibly due to maturational delay or degeneration [12].

In 2008, Dutch scientists evaluated histologic changes in a fetal sheep model of bladder exstrophy and fetal repair of this defect [13] by means of a molecularly defined dual-layer collagen biomatrix. The bladders of 12 fetal lambs were opened by incision; in six lambs, the bladder was kept open by sutures to mimic uncorrected bladder exstrophy. In the other six lambs, a dual layer collagen biomatrix was sutured into the bladder wall and the abdominal wall closed. After birth, the bladders were evaluated with respect to histological changes. The group of lambs with exstrophic bladders showed remarkable histologic similarity to the exstrophic bladders of humans, such as inflammatory and maturational changes in the entire bladder wall, including the detrusor muscle. Unfortunately, the S100 staining did not sufficiently differentiate between nerve fibers and smooth muscle cells to evaluate the content of nerve fibers [13].

Our findings are comparable to previous studies that evaluated the innervation pattern of the exstrophic bladder wall. Although the sensory and sympathetic innervation pattern in the unclosed exstrophic detrusor muscle appears to be similar or even reduced to that of normal bladders, more studies are necessary to evaluate the development of innervation in the bladder wall.

This study has some limitations. First, our cohort only included patients with unclosed BE undergoing delayed bladder closure. Further studies should include samples of newborns undergoing immediate closure. Second, the median age of the group of patients with BE and the control group differed considerably, so early development of arrest of nerve growth may have played a certain role. Third, the small sample size can influence statistical power and generalizability. The p-value is affected by the sample size [22, 23] and the probability of a Type II error increases with small sample sizes [24]. Thus, it is recommended that clinical considerations not be based solely on p values. Instead, other contextual aspects should be considered, such as the distribution of the density of the different immunohistochemical parameters to obtain tendencies. However, the number of specimens in our study represents a large sample size for this rare entity. Last, generalizing observations from a randomly chosen small sample that may not be representative of the entire population may also be problematic [25].

Despite these limitations, to our knowledge, this study is unique in assessing the potential role of NGF as a neural modulator for innervation of the exstrophic bladder wall and could provide consecutive patients referred for delayed primary closure and a large cohort for this rare entity. We believe that this investigation can provide a platform for future studies.

## Conclusions

This study assessed the innervation pattern of the detrusor muscle in unclosed exstrophic bladders with known overexpression of NGF in the urothelium. Although the comparison of neuronal markers between patients with BE and patients with VUR did not show significant differences, the density of the markers showed a lower density of nerve fibers in the detrusor muscle of patients with BE. It remains unclear whether these findings represent an early development state or an expression of a general disorder of the innervation pattern in exstrophic bladders.

More studies on the innervation pattern including muscarine receptors in closed bladders and the long-term development of detrusor function are necessary to evaluate the possible impact of NGF as a modulator of innervation of the bladder wall and its role in clinical outcome.

## Data Availability

The dataset generated during and/or analysed during the current study are avaiable from the corresponding author on reasonable request.

## Abbreviations

Anti-CGRP: Anti-calcitonin gene-related peptide
Anti-NF200: Anti-neurofilament 200
Anti-TH: Anti-tyrosine hydroxylase
BE: Classic bladder exstrophy
BEEC: Bladder-exstrophy-epispadias complex
M receptor: Muscarinic receptor
NGF: Nerve growth factor
NGFR p75: Nerve growth factor p75
NPY: Neuropeptide Y
SP: Substance P
VIP: Vasoactive intestinal polypeptide
VUR: Vesicoureteral reflux
